# Accurate Prediction of a Quantitative Trait Using the Genes Controlling the Trait for Gene-Based Breeding in Cotton

**DOI:** 10.3389/fpls.2020.583277

**Published:** 2020-11-09

**Authors:** Yun-Hua Liu, Yang Xu, Meiping Zhang, Yanru Cui, Sing-Hoi Sze, C. Wayne Smith, Shizhong Xu, Hong-Bin Zhang

**Affiliations:** ^1^Department of Soil and Crop Sciences, Texas A&M University, College Station, TX, United States; ^2^Botany and Plant Sciences, University of California, Riverside, Riverside, CA, United States; ^3^Department of Computer Science and Engineering and Department of Biochemistry and Biophysics, Texas A&M University, College Station, TX, United States

**Keywords:** quantitative trait, phenotype prediction, fiber length, fiber length gene, genic SNP, gene expression, *Gossypium*

## Abstract

Accurate phenotype prediction of quantitative traits is paramount to enhanced plant research and breeding. Here, we report the accurate prediction of cotton fiber length, a typical quantitative trait, using 474 cotton (*Gossypium* ssp.) fiber length (*GFL*) genes and nine prediction models. When the SNPs/InDels contained in 226 of the *GFL* genes or the expressions of all 474 *GFL* genes was used for fiber length prediction, a prediction accuracy of *r* = 0.83 was obtained, approaching the maximally possible prediction accuracy of a quantitative trait. This has improved by 116%, the prediction accuracies of the fiber length thus far achieved for genomic selection using genome-wide random DNA markers. Moreover, analysis of the *GFL* genes identified 125 of the *GFL* genes that are key to accurate prediction of fiber length, with which a prediction accuracy similar to that of all 474 *GFL* genes was obtained. The fiber lengths of the plants predicted with expressions of the 125 key *GFL* genes were significantly correlated with those predicted with the SNPs/InDels of the above 226 SNP/InDel-containing *GFL* genes (*r* = 0.892, *P* = 0.000). The prediction accuracies of fiber length using both genic datasets were highly consistent across environments or generations. Finally, we found that a training population consisting of 100–120 plants was sufficient to train a model for accurate prediction of a quantitative trait using the genes controlling the trait. Therefore, the genes controlling a quantitative trait are capable of accurately predicting its phenotype, thereby dramatically improving the ability, accuracy, and efficiency of phenotype prediction and promoting gene-based breeding in cotton and other species.

## Introduction

Many traits of agricultural and medical importance, such as crop yield, livestock productivity and human diseases, are known as quantitative traits that are each controlled by numerous genes. Therefore, it has been one of the principle aims and interests of current molecular and genomic research to accurately predict the phenotypes of quantitative traits for progeny selection using omic data, thereby enhancing the ability, accuracy, and efficiency of breeding in crop plants (Crossa et al., 2010, 2013; De Los Campos et al., 2010b; Heffner et al., 2011a,b; González-Camacho et al., 2012; Gouy et al., 2013; Desta and Ortiz, 2014; Xu et al., 2014, 2016; Beyene et al., 2015; Dan et al., 2016) and livestock (Meuwissen et al., 2001; Daetwyler et al., 2012; Morota et al., 2014), and medicine in humans (Khan et al., 2001; Lee et al., 2008; De Los Campos et al., 2010a; Speed and Balding, 2014; Weissbrod et al., 2016). This has been known as genomic selection (GS) in crop plant and livestock breeding (Meuwissen et al., 2001; Desta and Ortiz, 2014) and as genomic medicine in humans (De Los Campos et al., 2010a). A so-called training population, usually a subpopulation of individuals randomly selected from a targeted breeding population, is both phenotyped and genotyped, and used to train and validate a statistical prediction model. The utility and efficiency of the trained model for phenotype prediction of the objective trait are often estimated by prediction accuracy presented by Pearson’s correlation coefficient between observed and predicted phenotypes. The remaining individuals of the targeted population are genotyped only and their genetic values or phenotypes of the objective trait are then estimated using the trained and validated prediction model. The predicted phenotypes of the trait for the individuals of the targeted population are finally used to make decision for progeny selection in crop plant and livestock breeding, and for medicine practice in humans (De Los Campos et al., 2010a).

Because of their polygenic controls and sensitivity to varying environments, accurate prediction of quantitative traits is very challenging. Initially, genome-wide DNA markers were used to predict the phenotypes of quantitative traits (Meuwissen et al., 2001; Lee et al., 2008; Crossa et al., 2010, 2013; De Los Campos et al., 2010b; Heffner et al., 2011a,b; Daetwyler et al., 2012; González-Camacho et al., 2012; Gouy et al., 2013; Morota et al., 2014; Speed and Balding, 2014; Xu et al., 2014; Beyene et al., 2015; Weissbrod et al., 2016). Then, genome-wide gene expressions (Takagi et al., 2014; Xu et al., 2016) and genome-wide metabolites (Dan et al., 2016; Xu et al., 2016) have been used to improve the prediction accuracy of the trait phenotype. Attempts have been also made to improve the prediction accuracy of quantitative traits by increasing training population size, from hundreds to thousands of lines, and/or increasing the omic dataset size, from hundreds to millions of features (Lee et al., 2008; González-Camacho et al., 2012; Speed and Balding, 2014; Xu et al., 2016). Furthermore, approximately 20 statistical multiple regression models, including parametric and non-parametric, have been tested for the phenotype prediction of quantitative traits using the omic features (Desta and Ortiz, 2014; Speed and Balding, 2014; Weissbrod et al., 2016). These efforts have improved the prediction accuracy of quantitative traits, but the prediction accuracy still remains relatively low for the quantitative traits thus far investigated. The lower prediction accuracy and increased cost for phenotype prediction, due to the increased numbers of DNA markers and/or training population size, have substantially influenced applications of GS in practical breeding in crop plants and livestock. Most importantly, plant or livestock breeding usually consists of three parts: parent selection, cross design, and progeny selection. GS is effective for progeny selection, but it is ineffective for parent selection and cross design, while both are crucial to success of plant or livestock breeding.

Therefore, Zhang et al. (2020a), for the first time worldwide, proposed a novel molecular breeding technology, designated gene-based breeding (GBB), and demonstrated its utility and efficiency for enhanced breeding for maize grain yield. GBB is designed to develop new varieties by design by making full use of the genes controlling the objective trait(s), especially the number of their favorable alleles (NFAs), their SNPs/InDels as DNA markers and/or their expression abundances as omic features, through the entire breeding process, including parent selection, cross design, and progeny selection. Zhang et al. (2020a) showed that the prediction accuracy of maize grain yield using either of these three datasets of the grain yield genes for GBB was over 60% more accurate and several-fold more cost-efficient than those with genome-wide random SNPs. When the phenotypes of grain yield predicted with two or all of three datasets of the genes were jointly used for progeny selection, the top 10% plants selected using the predicted grain yields were completely consistent with those selected based on the grain yields of the plants determined by replicated field trials. Therefore, their results showed that GBB is promising to substantially continue crop improvement. Nevertheless, additional research is needed to test the utility and efficiency of GBB for different traits in different species and to optimize it for enhanced breeding of different crops and livestock.

In the present study, we explored the ability, utility, and efficiency of the genes significantly contributing to quantitative traits for prediction of their phenotypes using fiber length as the objective trait in cotton. Cotton, including *Gossypium hirsutum* L. (Upland cotton) and *Gossypium barbadense* L. (Sea Island cotton), is the world’s leading textile fiber crop and an important oilseed crop. Fiber length is a typical quantitative trait and also one of the economically most important fiber quality traits for the textile industry and cotton fiber produce. We previously cloned 474 *GFL* (*Gossypium* fiber length) genes significantly contributing to fiber length (upper half mean length, UHML) and estimated their effects on fiber length (Liu, 2014). In this study, we investigated the phenotype prediction ability and efficiency of cotton fiber length for gene-based breeding using these *GFL* genes. We also discussed the applicability of the concepts and methods obtained in the present study to development of GBB for enhanced breeding in other crops and livestock of agricultural importance.

## Materials and Methods

### Plant Materials and Fiber Length Phenotyping

**FIGURE 1:**
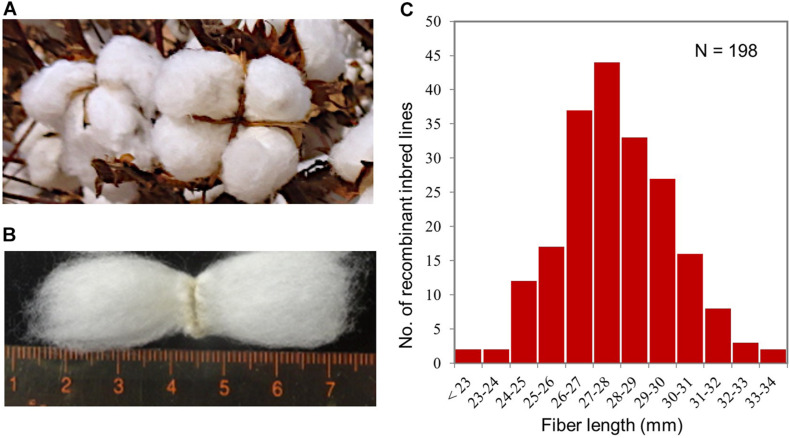
Field trial of the RIL population for fiber length phenotyping. **(A)** Matured fiber bolls used for fiber length phenotyping. **(B)** Fiber lengths. **(C)** Variation of fiber length in UHML, measured by high-volume instrumentation, in the RIL population showing that fiber length is a typical quantitative trait. The fiber length data were collected from the 2011 field trial and the mean fiber lengths of three replicates.

One hundred ninety-eight recombinant inbred lines (RILs) at F_7_, F_8_, and F_9_ generations derived by the single-seed descent method from a cross of TAM 94L-25 (*G. hirsutum*) x NMSI 1331 (*G. barbadense*) were used for this study. These RILs and their parents were grown at the Texas A&M AgriLife Research Farm near College Station, TX, United States, in 2009 (F_7_), 2010 (F_8_), and 2011 (F_9_) to phenotype their fiber lengths. The 2010 and 2011 field trials were performed in a randomized complete block design, with three replicates, while the 2009 trial only included a single five-plant plot per line, with no replication, because it was used for seed production for the 2010 and 2011 trials. The field practices followed those used for standard cotton breeding trials in our cotton breeding program. When the fiber bolls completely ripened (Figure 1A), they were hand-harvested from entire plots and ginned. A sample of the fibers from each line was used to measure its fiber length (Figure 1B), presented as upper half mean length (UHML), using High-Volume Instrumentation (HVI) at Fiber and Biopolymer Research Institute, Texas Tech University, Lubbock, TX, United States.

The mean fiber length of each line was calculated from those of the three replicates for each of the 2010 and 2011 trials (Figure 1C). The fiber length of the 2009 trial was from single five-plant entry. The broad sense heritability (*H*^2^) of fiber length was estimated separately for the 2010 and 2011 trials by subtracting the mean fiber length variance of the two parents among their entries (*n* = 33 for each parent) [σ^2^*_*e*_* = (σ*${}_{p\,1}^{2}$* + σ^2^*_*p2*_)/*2] from the fiber length variance of the 198 RILs (σ^2^*_*p*_*) and then dividing by the fiber length variance of the 198 RILs (σ^2^*_*p*_*).

### Genes

#### *GFL* Genes

The 474 *GFL* genes were previously cloned by our laboratory and coded from 001 through 474 (Liu, 2014) were used for this study (Supplementary Table S1A; NCBI GenBank accession numbers: MW082098-MW082571). These 474 *GFL* genes included 17 of the 18 published fiber length genes (Supplementary Tables S2, S3; Zhang et al., 2020b). Liu (2014) showed that each of these *GFL* genes had an effect on fiber length varying from 2.6% to 7.9%, with 88.6% of them significantly decreasing and 11.4% significantly increasing fiber length, when activated or up-regulated (Supplementary Table S1A). Network analysis showed that for 19 of these 474 *GFL* genes, variation of their edge numbers in the *GFL* network was significantly associated with fiber length (Supplementary Table S1B) (Liu, 2014; for more related information, see Zhang et al., 2020b).

#### Published Fiber Length Genes

A literature search was conducted as of December 2014 and found that a total of 18 fiber length genes were cloned from cotton using different gene cloning methods, including gene expression repression (RNAi or antisense) and gene overexpression (Supplementary Table S2; Zhang et al., 2020b). These 18 published fiber length genes were used as the positive control to test the ability of the *GFL* genes to predict the phenotype of fiber length in this study.

#### Randomly Selected Cotton Unknown Non-474 *GFL* Genes

A cotton database consisting of 79,708 transcripts of developing fibers sampled on the 10^th^ day of post-anthesis (10-dpa fibers) (Zhang et al., 2019) were used for sampling the randomly selected cotton unknown non-474 *GFL* genes used as the negative control in this study.

### Gene Transcript Expression Profiling and Gene Transcript Expression Dataset Construction

The sequences of the TAM 94L-25 transcripts expressed in 10-dpa fibers (Zhang et al., 2019), including those of the 474 *GFL* genes, were used as the reference to determine the expression profiles of the targeted transcripts of the *GFL* genes in the 10-dpa developing fibers of each line. Because a plant gene may be alternatively spliced into multiple transcripts, with each transcript likely being translated into different proteins having different biological functions (Syed et al., 2012; Zhang et al., 2019), the expression abundances of only the transcripts of the *GFL* genes that are responsible for fiber length (Zhang et al., 2020b) were quantified as predictors for phenotype prediction of fiber length in this study. The targeted transcript expression abundance of each *GFL* gene in a line was quantified with the RNA-seq 100-nucleotide clean reads using the RSEM software (Li and Dewey, 2011) bundled with the Trinity software (Grabherr et al., 2011; Haas et al., 2013) and presented as Transcripts Per Million mapped reads (TPM) (Supplementary Table S4).

### *GFL* SNP/InDel Genotyping and SNP/InDel Dataset Construction

We previously sequenced all the genes expressed in 10-dpa developing fibers of the cotton population from the 2011 trial (Liu, 2014; Zhang et al., 2019). In this study, we first identified the single nucleotide polymorphisms (SNPs) and/or nucleotide insertions/deletions (InDels) of all the expressed genes using the RNA-seq 100 nucleotide clean reads and SAMtools (Li et al., 2009; Li, 2011). The cotton acc. TM-1 genome (Zhang et al., 2015) was used as the reference. Only the SNPs or InDels identified at the same position in the two parents, TAM 94L-25 and NMSI 1331, and two or more lines were used for further analysis. Since the transcript assemblies of the expressed genes had an average length of 778 bp (Liu, 2014), the probability that the two parents and two RILs had an SNP or InDel at the same position by chance, such as sequencing, base calling, and/or transcript assembly errors, would be close to zero [*P* = (1/778)^4^ = 2.7E-12]. This filtration excluded almost all SNPs or InDels, if not all, resulted from sequencing, base calling, and/or transcript assembly errors from this study.

Then, we extracted the SNPs and/or InDels (hereafter, SNPs/InDels) contained in the *GFL* genes. To identify the SNPs/InDels of the *GFL* genes that significantly influenced fiber length, we conducted association analysis between the *GFL* genic SNPs/InDels and fiber length using the single marker analysis method for QTL mapping (Liu, 1997). Given that cotton has a genome size of 2,450 Mb/1C, the probability of the *GFL* genic SNPs/InDels linked to a gene controlling fiber length within an interval of 10 Mb, if they were the SNPs/InDels contained in the *GFL* genes, would be extremely low [(10/2,450)^2^ = 1.67E-05]. Therefore, the association of a *GFL* genic SNP/InDel with fiber length indicated that the SNP/InDel of the *GFL* gene highly likely had a significant effect on fiber length. Therefore, only the SNPs/InDels contained in the *GFL* genes significantly influenced fiber length (*P* ≤ 0.05) were selected and used as DNA markers for this study. These genes were defined in this article as the SNP/InDel-containing *GFL* genes. Furthermore, the *GFL* genic SNPs were verified by allele-specific PCR using the genomic DNAs of four cotton genotypes, including the two parents of the cotton population, as templates (Gaudet et al., 2007).

For the construction of the *GFL* genic genotype dataset, their SNPs or InDels were scored as bi-allelic DNA markers, as those genome-wide SNPs used for prediction of phenotype for genomic selection. The homozygote for one allele was scored as “0,” the homozygote for the other allele scored as “2,” and their heterozygote scored as “1.” Because cotton is a frequently outcrossing species and the RIL population used in this study was developed in the field condition, with no bagged selfing pollination, heterozygotes for some plants were expected, even though the RILs at F_7_–F_9_ generation were used for this study.

### Fiber Length Prediction

Prediction of fiber length using the *GFL* genes was carried out with two genic datasets compiled above separately: (i) the SNPs or InDels contained in the SNP/InDel-containing *GFL* genes as DNA markers and (ii) the targeted transcript expressions of the *GFL* genes in 10-dpa developing fibers. Nine prediction models, including five parametric and four non-parametric models (Desta and Ortiz, 2014; Zhang et al., 2020b), that have been widely used for GS were used to predict fiber length using the *GFL* genes. The five parametric models were genomic best linear unbiased prediction (GBLUP) (VanRaden, 2008), least absolute shrinkage and selection operator (LASSO) (Tibshirani, 1996), partial least square (PLS) (Geladi and Kowalski, 1986), BayesA (González-Recio and Forni, 2011), and BayesB (González-Recio and Forni, 2011). The four non-parametric models were support vector machine using the radial basis function kernel (SVMRBF) (Maenhout et al., 2007), support vector machine using the polynomial kernel function (SVMPOLY) (Maenhout et al., 2007), random forest (RF) (Svetnik et al., 2003), and reproducing kernel Hilbert space regression (RKHS) (De Los Campos et al., 2010a). We tested these nine prediction models because some of them may not be well suited for these two datasets, while others may be well fitted for the prediction of fiber length using the datasets.

GBLUP was implemented in an R program (Xu et al., 2014); LASSO was implemented in the GlmNet/R program (Friedman et al., 2010); BayesA, BayesB, and RKHS were implemented in the BGLR package (Pérez and De Los Campos, 2014); SVMRBF and SVMPOLY were implemented in the kernlab R program (Karatzoglou et al., 2004); PLS was implemented using the pls R package (Mevik and Wehrens, 2007); and RF was implemented in an R program (Liaw and Wiener, 2018). Among the nine prediction models, several require tuning parameters, which were selected based on the 10-fold cross validation used for the prediction (see below). Parameter values that maximize the predictability (squared correlation between predicted and observed trait values) were chosen as the optimal values. The shrinkage parameter of LASSO was chosen in this way. For the PLS prediction, the number of components extracted was considered as a tuning parameter and was obtained *via* 10-fold cross validation also. For BayesA, BayesB, and RKHS, the number of iterations, burnIn and thin were set to 10000, 1000 and 10, respectively. For RKHS, a multi-kernel approach was used, as proposed by De Los Campos et al. (2010b), and the bandwidth parameter was set to {0.5, 2, 10}.

A 10-fold cross-validation scheme widely used for GS was used for the prediction of fiber length using the *GFL* genes. The 10-fold cross validation scheme was described in our previous study (Zhang et al., 2020a), with each subset consisting of 19 or 20 RILs and 100 replications.

### Statistical Analysis

The statistical analyses, including the two-way ANOVA, Tukey’s HSD (honest significant difference), and parametric correlation tests, were performed using an R program and Microsoft Excel 2013. For the ANOVA and correlation tests, *P*-value was presented at a two-tailed significance, and for the Tukey’s HSD test, a confidence interval (CI) of 95% was applied.

## Results

### Variation of Cotton Fiber Length, and Transcript Expression Variation and SNPs/InDels of the *GFL* Genes

Phenotype analysis confirmed that the fiber length trait (Figures 1A,B) under this study exhibited a normal distribution (Figure 1C), the variation of a typical quantitative trait, for the field trials through all three years (2009, 2010, and 2011) and all three generations (F_7_, F_8_, and F_9_) among the 198 RILs of the population studied. The fiber lengths of the population from the 2009, 2010, and 2011 trials varied from 23.0 to 34.6, 23.1 mm to 35.8 mm, and from 23.1 mm to 34.8 mm, respectively. Figure 1C shows the variation of fiber length determined through the 2011 field trial. The Pearson’s correlation coefficients (*r*) of the fiber length phenotypes between the three replicates of the 2010 and 2011 trials were 0.80–0.85 (*N* = 164, *P* = 0.000) and 0.76 (*N* = 198, *P* = 0.000), respectively. The Pearson’s correlation coefficients (*r*) of the fiber length phenotypes between the 2009, 2010, and 2011 trials were 0.67–0.91 (*N* = 164 or 198, *P* = 0.000), even though the weather of the trial location in 2011 was unusual hot and drought, which was quite different from those normal weathers in 2010 and 2009. The broad sense heritability of the fiber length was *H*^2^ = 0.90 and 0.83 for 2010 and 2011, respectively, which were similar to those previously reported (Ulloa, 2006; Khan et al., 2010). We were unable to calculate the *H*^2^ for 2009 because there was no replication for the parents for the 2009 trial to estimate the environmental variance (σ*${}_{e}^{2}$*).

SNP/InDel analysis revealed that 400 of the 474 *GFL* genes contained one or more SNPs/InDels and 74 had no SNPs/InDels for the population. The 400 *GFL* genes had a total of 10,766 SNPs/InDels, with an average of 26.9 SNPs/InDels per gene. Gene mutation effect analysis showed that 740 (6.9%) of the SNPs/InDels contained in 226 of the 400 *GFL* genes, with an average of 3.2 SNPs/InDels per gene, significantly increased or decreased fiber length (*P* ≤ 0.05) of the RILs (Supplementary Tables S1C, S6) by 2.1% to 22.6%. The multiple SNPs/InDels per *GFL* gene suggested that there are multiple alleles for a *GFL* gene, if each of its SNPs/InDels was considered to be biallelic. The number of SNPs that significantly influenced fiber length was expected, because a vast majority of the SNPs contained in protein-coding genes are known to be synonymous, not leading to protein sequence change and likely having no biological effects (Graur and Li, 2000). Furthermore, we randomly selected 20 SNPs from the 740 *GFL* SNPs/InDels, with one SNP from a *GFL* gene, and analyzed them by allele-specific PCR using the genomic DNAs of four cotton genotypes as templates, including the two parents of the population used in this study. The result confirmed the existence of all 20 SNPs in the four genotypes, with the sizes of the PCR products as expected (Supplementary Figure S1), thus confirming the *GFL* genic SNPs identified. Therefore, these 226 *GFL* genes were hereafter defined as SNP/InDel-containing *GFL* genes and further used as DNA markers for phenotype prediction of fiber length.

The 474 *GFL* genes all expressed in 10-dpa developing fibers of the population, but their expressions varied by thousands fold, from 0.75 TPM to 23,601 TPM (Supplementary Table S4). The expression of each *GFL* gene also varied dramatically among the RILs of the population, with a coefficient of variance (CV%) of 18.5%–202.5%. The expressions of all 474 *GFL* genes exhibited quantitative variations, with approximately 60% showing normal distributions and approximately 40% having distributions biased to lower expressions. Correlation analysis showed that the expressions of all 474 *GFL* genes in 10-dpa developing fibers were significantly correlated with the variation of the fiber length in the population (*P* ≤ 0.05), which was consistent with the expression correlation of previously published fiber length genes (Supplementary Tables S2, S3) with the variation of fiber length (Zhang et al., 2020b). Therefore, both SNP/InDel and expression analyses further confirmed that the 474 *GFL* genes controlling fiber length.

### Predicting the Phenotype of Fiber Length Using the *GFL* Genes

We tested the utility and efficiency of the *GFL* genes for phenotype prediction of fiber length for enhanced cotton fiber length breeding through GBB, especially progeny selection in this study, using expression abundances and SNP/InDel genotypes of the *GFL* genes. We first trained and validated the nine prediction models using the fiber length data collected from the 2011 trial, because the RILs of the population from the 2011 trial were also genotyped using the expressions and SNPs/InDels of the *GFL* genes. Then, we tested the utility and efficiency of the trained prediction model selected above for phenotype prediction of fiber length for the 2009 (F_7_) and 2010 (F_8_) trials using the genotypic data from the 2011 trial.

#### Predicting the Phenotype of Fiber Length Using the Expressions of the *GFL* Genes

**FIGURE 2:**
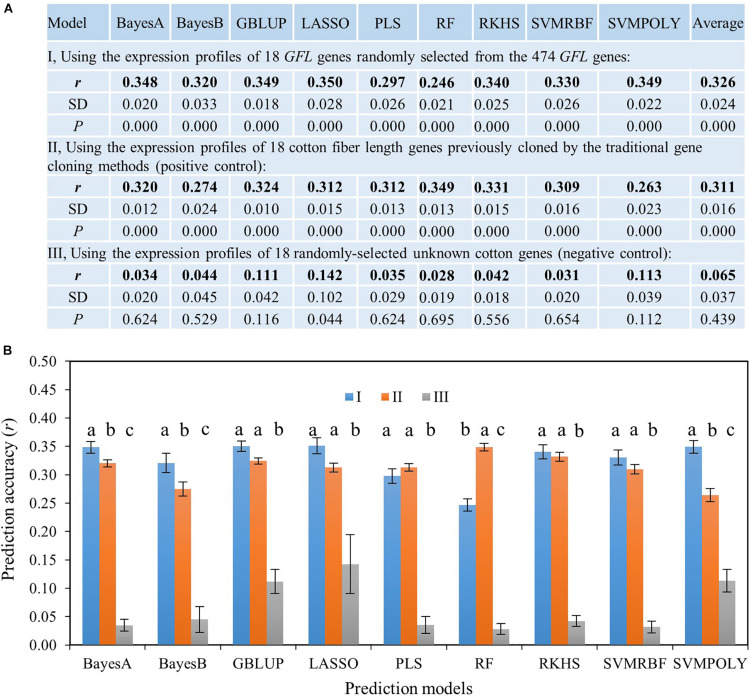
Ability of the *GFL* genes to predict the phenotype of fiber length using nine prediction models. **(A)** Ability of the *GFL* genes to predict the phenotype of fiber length using 18 *GFL* genes randomly selected from the 474 *GFL* genes. *r*, prediction accuracy presented by Pearson’s correlation coefficient between predicted and observed fiber lengths; SD, standard deviation for 100 replications. **(B)** Statistics of prediction accuracies between these three sets of genes described in **(A)** for fiber length using the Tukey’s HSD. I, 18 randomly-selected *GFL* genes. II, 18 published cotton fiber length genes (Supplementary Tables S2, S3); III, 18 randomly selected unknown cotton non-474 *GFL* genes. Different letters, significant at a confidence interval (CI) ≥ 95%; error bar, standard deviation for 100 replications. GBLUP, genomic best linear unbiased prediction; LASSO, least absolute shrinkage and selection operator; PLS, partial least square; SVMRBF, support vector machine using the radial basis function kernel; SVMPOLY, support vector machine using the polynomial kernel function; RF, random forest; RKHS, reproducing kernel Hilbert space regression (RKHS).

We first tested the ability of the *GFL* genes for predicting the phenotype of fiber length, in which the published fiber length genes previously cloned by different researchers using different gene cloning methods (Supplementary Tables S2, S3) were used as the positive control. Since only 18 published genes controlling cotton fiber length were previously cloned as of December 2014, the ability of the *GFL* genes to predict the phenotype of fiber length was first evaluated using only 18 *GFL* genes randomly selected from these 474 *GFL* genes. These 18 published fiber length genes were used as the positive control, and 18 randomly selected unknown cotton genes were used as the negative control. Nine prediction models widely used for prediction of quantitative traits for GS and the expressions of the 18 *GFL* genes (Supplementary Table S4), 18 previously published fiber length genes (Supplementary Table S2) and 18 randomly selected unknown genes were used to predict fiber length, respectively. Results showed that only the randomly selected *GFL* genes and the published fiber length genes could predict the phenotype of fiber length, with a prediction accuracy of *r* = 0.246–0.350 (*P* = 0.000). The randomly selected unknown cotton genes could not predict the fiber length (*r* = 0.028–0.142, *P* > 0.05 for all nine prediction models, except for LASSO that had *P* = 0.044) (Figure 2A). Tukey’s HSD test showed that the *GFL* genes had a similar prediction ability of fiber length to the published fiber length genes for five of the nine prediction models tested (confidence interval, CI < 95%), a higher prediction ability of fiber length than the published fiber length genes for three of the models, BayesA, BayesB, and SVMPOLY (CI ≥ 95%), and a lower prediction ability of fiber length than the published fiber length genes for only one of the nine models, RF (CI ≥ 95%). Both the *GFL* genes and the published fiber length genes had significantly higher prediction abilities than the randomly selected unknown genes for all nine prediction models (Figure 2B). These results indicated that the *GFL* genes had similar or better abilities to predict the fiber length than the published fiber length genes, thus verifying the contributions of the *GFL* genes to fiber length and their utility and efficiency to predict the phenotype of the objective trait.

**FIGURE 3:**
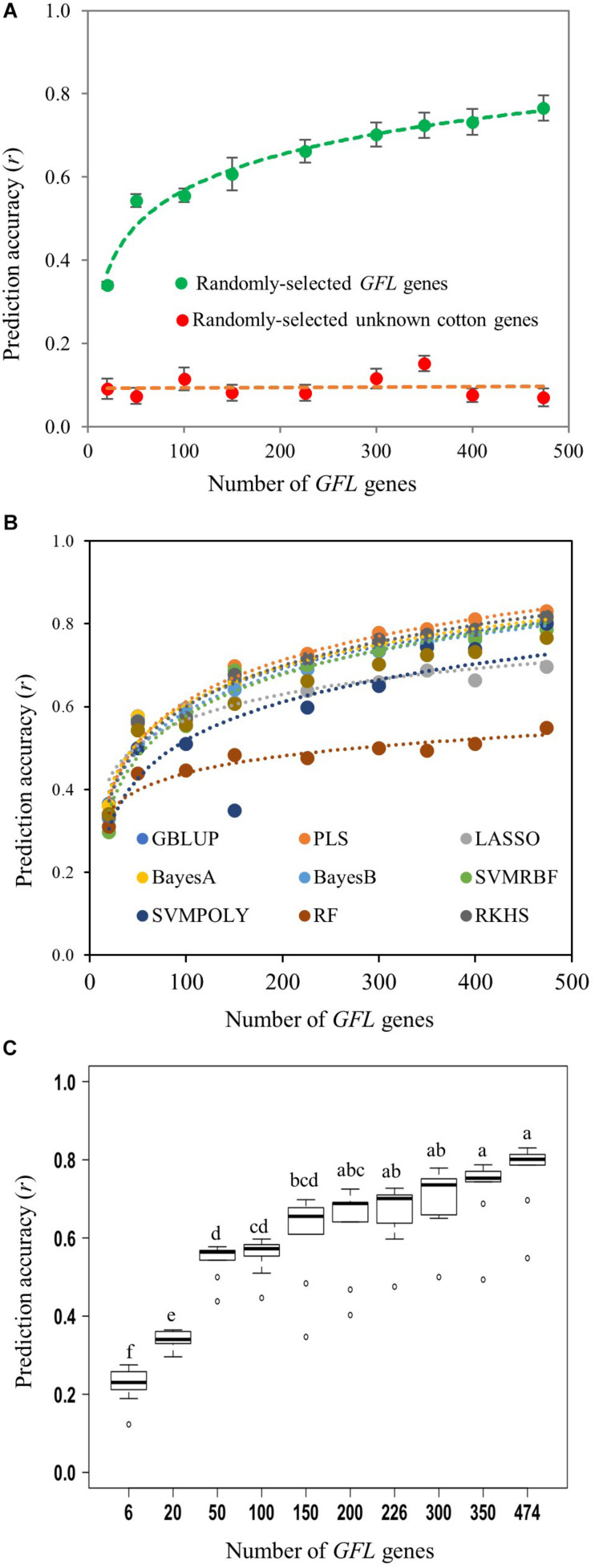
Prediction of fiber length with different numbers of randomly selected *GFL* genes and nine prediction models using expression profiles. **(A)** Mean prediction accuracy of fiber length with the *GFL* genes using the nine prediction models. A series of numbers of the 474 *GFL* genes ranging from 6 to 474 were tested using the same numbers of randomly selected unknown cotton non-474 *GFL* genes as the negative control (Supplementary Table S5). For prediction models, see Figure 2. **(B)** Prediction accuracy of fiber length with the *GFL* genes using different prediction models. **(C)** Statistics of the mean prediction accuracies between different numbers of the *GFL* genes predicted by the nine prediction models using the Tukey’s HSD. Different letters, significant at CI ≥ 95%; same letter, not significant at CI ≥ 95%.

Then, we further confirmed the ability of the *GFL* genes to predict the fiber length using a series of numbers of the randomly selected *GFL* genes sampled by bootstrap sampling, from 6 to all 474 (Figure 3 and Supplementary Table S5). The experiment had ten bootstrap selections for each number of genes. As expected, all sets of the randomly selected *GFL* genes tested, no matter how many *GFL* genes there were in the selection, from 6 to 474, and which of the prediction models was used, were able to predict the fiber length (*P* = 0.010 for 6 *GFL* genes and *P* = 0.000 for all selections of genes with a number of *GFL* genes greater than 6). Again, none of the randomly selected unknown gene selections, regardless of how many there were in the selection, from 6 to 474, and which of the nine prediction models was used, could predict the fiber length (*P* = 0.091–0.505) (Figure 3A and Supplementary Table S5). Furthermore, as the number of the *GFL* genes used for the prediction increased, the prediction accuracy of fiber length increased (Figures 3A,B). When 200 or more of the *GFL* genes were used, the prediction accuracy plateaued (Figure 3C). Comparative analysis showed that the prediction models, PLS, BayesA, and RKHS, best predicted the phenotype of fiber length among the nine prediction models tested, with a prediction accuracy of *r* = 0.830, 0.817, and 0.814, respectively, when all 474 *GFL* genes were used (Figure 3B and Supplementary Table S5). In contrast, the prediction accuracies of the randomly-selected unknown gene sets remained non-significant, low, and consistent, for all of the randomly-selected cotton unknown gene selections, from 6 to 474 (Figure 3A and Supplementary Table S5). These results further confirmed the ability, utility, and efficiency of the *GFL* genes for accurate prediction of fiber length.

#### Prediction of Fiber Length Using the SNPs/InDels of the *GFL* Genes as DNA Markers

**FIGURE 4:**
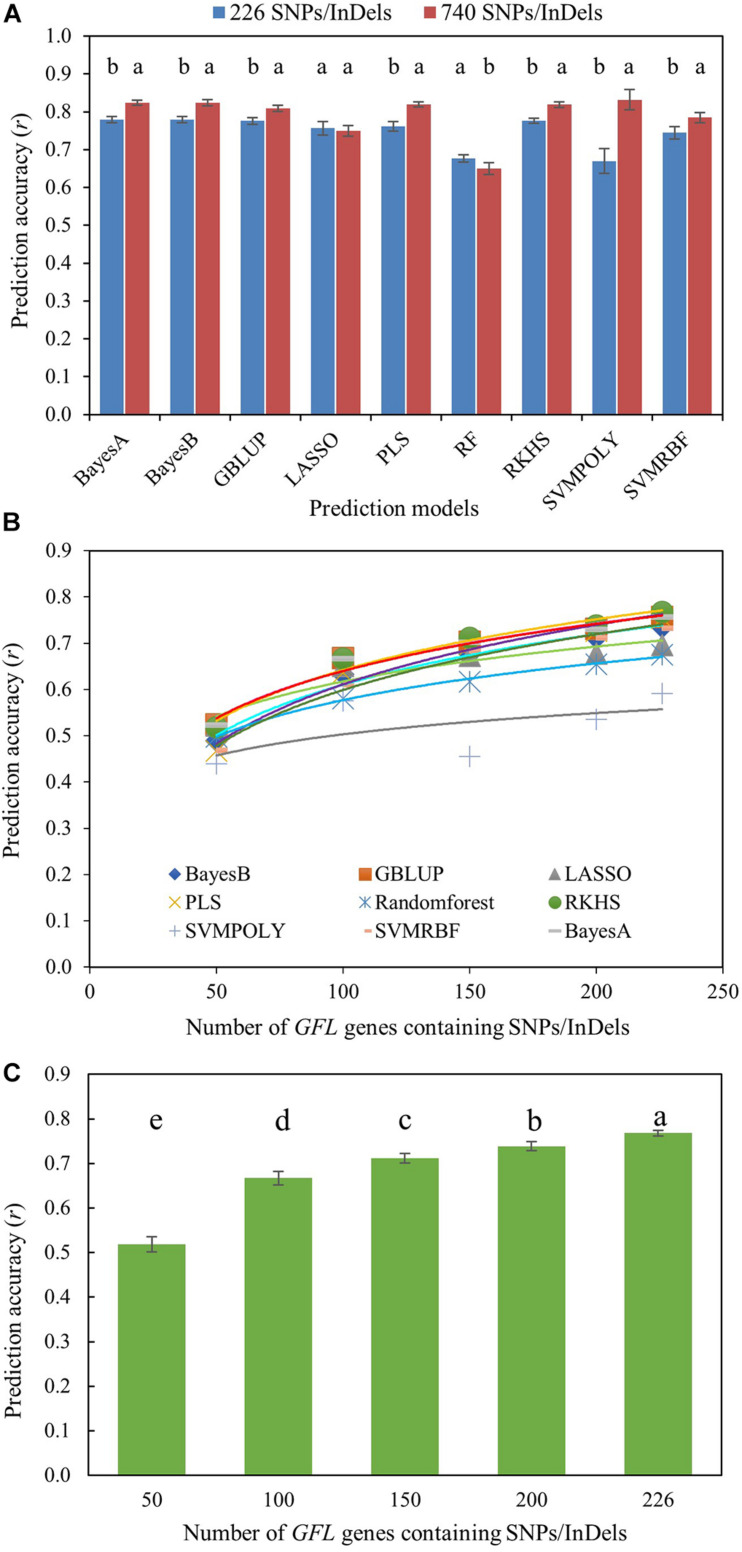
Prediction of fiber length with the *GFL* SNPs/InDels as DNA markers using nine prediction models. **(A)** Prediction accuracy of fiber length using the genotypes of all 740 *GFL* SNPs/InDels (Supplementary Table S6) versus a selection of 226 *GFL* SNPs/InDels that had the largest effects on fiber length, with only one SNP/InDel per gene (Supplementary Table S7). Different letters, significant at CI ≥ 95%; same letter, not significant at CI ≥ 95%; error bar, standard deviation for 100 replications. **(B)** Prediction accuracy of fiber length with the selection of the 226 *GFL* SNPs/InDels (Supplementary Table S7) using different prediction models. **(C)** Prediction of fiber length with different numbers of the 226 *GFL* SNPs/InDels (Supplementary Table S7) using the RKHS model. Different letters, significant at CI ≥ 95%; error bar, standard deviation.

Moreover, we further tested the ability, utility, and efficiency of the *GFL* genes in predicting the phenotype of fiber length using the 226 SNP/InDel-containing *GFL* genes (Supplementary Table S1C). The SNPs or InDels contained in the 226 SNP/InDel-containing *GFL* genes were only used as DNA markers (Supplementary Tables S6, S7), as those DNA markers used for GS, with no effect of the *GFL* genes on fiber length considered, for the prediction. We first compared the prediction accuracy of fiber length using all 740 SNPs/InDels contained in the 226 *GFL* genes (Supplementary Table S6) and a selection of the 740 genic SNPs/InDels, with only one SNP/InDel that had the largest effect on fiber length per *GFL* gene (Supplementary Table S7). As expected, the 740 *GFL* SNPs/InDels better predicted the phenotype of fiber length, with a prediction accuracy varying from *r* = 0.650 (*P* = 0.000) for the RF model to *r* = 0.832 (*P* = 0.000) for the SVMRBF model, than the selection of the 226 *GFL* SNPs/InDels, with a prediction accuracy varying from *r* = 0.671 (*P* = 0.000) for the SVMPOLY model to *r* = 0.779 (*P* = 0.000) for the BaysA, BayesB, GBLUP, or RKHS model, in seven of the nine prediction models. The 740 *GFL* SNPs/InDels had a similar to or lower prediction accuracy than the selection of the 226 *GFL* SNPs/InDels for the LASSO and RF models (Figure 4A).

However, if the selection of 226 SNPs/InDels was used for the prediction, although the prediction accuracy would be slightly lower, the cost of genotyping for the prediction would be reduced by 2.3-fold. Therefore, we further tested the prediction accuracies of different numbers of the SNPs/InDels selected from the 226 *GFL* SNPs/InDels for the phenotype of fiber length. Overall, the RKHS model showed the best prediction results of fiber length among the nine models (Figure 4B), and as more of the 226 *GFL* SNPs/InDels were used, a more accurate prediction of fiber length was obtained (Figure 4C). The fiber lengths of the cotton lines were predicted at an accuracy of *r* = 0.783 (*P* = 0.000), when all the 266 *GFL* SNPs/InDels were used with the RKHS model.

In comparison, the prediction accuracies of fiber length using all 740 SNPs/InDels contained in 226 *GFL* genes were essentially the same high as the prediction accuracies of fiber length using the expressions of all 474 *GFL* genes, thus demonstrating the ability, utility and efficiency of the *GFL* genes in phenotype prediction of fiber length for progeny selection.

### Identification of the Key *GFL* Genes to Phenotype Prediction of Fiber Length for Progeny Selection

The above experiments indicated that the *GFL* genes were able to accurately predict the fiber length with either *GFL* expression abundances in 10-dpa developing fibers or *GFL* genic SNPs/InDels as DNA markers. The question was whether the *GFL* genes equally contributed to the phenotype prediction of fiber length. If not, whether a subset of the *GFL* genes, defined herein the key *GFL* genes, selected from the 474 *GFL* genes could predict the phenotype of fiber length as accurate as all 474 *GFL* genes for progeny selection. Therefore, we tested the ability and efficiency of the *GFL* genes according to their roles in the *GFL* network (Liu, 2014; Supplementary Table S1B), the effects of their SNP/InDel mutations on fiber length (Supplementary Table S1C), or their effects on fiber length (Liu, 2014; Supplementary Table S1A). The *GFL* genes randomly selected from the 474 *GFL* genes were used as the control. The expression abundances of the selected *GFL* genes were used for the prediction. Results showed that both the roles of the *GFL* genes in the *GFL* network (Supplementary Figure S2A) and their effects on fiber length (Supplementary Figure S2C) increased the ability of the genes to predict fiber length, but the effects of SNP/InDel mutations of the *GFL* genes on fiber length (Supplementary Figure S2B) decreased the ability of the genes to predict fiber length (CI ≥ 95%). Since the effects of the *GFL* genes on fiber length had a larger increase than their roles in the *GFL* network for phenotype prediction of fiber length, the subset of the 226 *GFL* genes consisting of all 54 positively effective *GFL* genes, 59 smallest negatively effective *GFL* genes, and 113 largest negatively effective *GFL* genes (Subset X, Supplementary Figure S2C) was selected for further analysis (Supplementary Table S1A).

**FIGURE 5:**
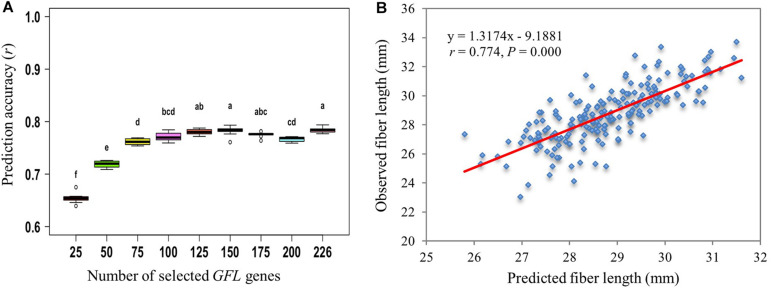
Prediction of fiber length using the 226 *GFL* genes selected according to their effects on fiber length (Subset X, Supplementary Figure S2C). **(A)** Prediction of fiber length using different numbers of the 226 selected *GFL* genes and the SVMRBF model. Different letters, significant at CI ≥ 95%; same letter, not significant at CI ≥ 95%; error bar, standard deviation for 100 replications. **(B)** Prediction of fiber length with the 125 *GFL* genes selected from the 226 *GFL* genes (Supplementary Table S8) using the SVMRBF model.

Furthermore, we predicted the phenotype of fiber length using different numbers of *GFL* genes randomly selected from the subset of 226 *GFL* genes above (Subset X, Supplementary Figure S2C). When 125 or more of the *GFL* gene subset were used, the prediction accuracy of fiber length plateaued for eight of the nine prediction models and the SVMRBF model best predicted the phenotype of fiber length using these numbers of the selected *GFL* genes (Figure 5A and Supplementary Figure S3). Therefore, a subset of 125 *GFL* genes were identified from the 226 selected *GFL* genes for phenotype prediction of fiber length using expression profiles in 10-dpa developing fibers (Supplementary Table S8). These 125 *GFL* genes were herein defined the key *GFL* genes to phenotype prediction of fiber length for progeny selection. When the 125 key *GFL* genes were used, the prediction accuracy of fiber length approached *r* = 0.774 (*P* = 0.000) (Figure 5B), suggesting that they were well suited for accurate prediction of fiber length and therefore, could be used for progeny selection in a breeding program. Comparative analysis showed that the prediction results of these 125 key *GFL* genes were significantly correlated with those predicted with all 474 *GFL* genes (*r* = 0.888, *P* = 0.000; Supplementary Figure S4). The fiber lengths predicted with the expression of the 125 key *GFL* genes were also significantly correlated with those predicted using the 226 SNPs/InDels contained in the 226 *GFL* genes (*r* = 0.892, *P* = 0.000).

### Prediction of Fiber Length Using the *GFL* Genes Across Years or Generations

To further explore the ability, utility, and efficiency of the *GFL* genes for fiber length prediction, we examined the prediction accuracy of fiber length for the RILs across years or environments (generations) using the two datasets of the selected *GFL* genes genotyped from the 2011 (F_9_) trial only and the fiber lengths phenotyped in 2009 (F_7_), 2010 (F_8_), and 2011 (F_9_), respectively. The result showed that the *GFL* genes genotyped in the 2011 (F_9_) trial could also predict the fiber length of the RILs grown in 2010 (F_8_) at a prediction accuracy similar to that achieved from the 2011 trial that was used for genotyping the genes using either of the two genic datasets, 125 key *GFL* expressions or 226 *GFL* SNPs/InDels as DNA markers. However, the prediction accuracy of fiber length for the RILs grown in 2009 (F_7_) was slightly lower than those achieved for the RILs in 2010 and 2011 (Table 1). Since the 2009 trial had no replication (those of 2010 and 2011 had three replications) and the prediction accuracy was determined by Pearson’s correlation coefficient between the predicted and observed phenotypes, the reduced prediction accuracy for 2009 could be more likely attributed to the fiber length phenotyping accuracy rather than the gene x environment interactions. These results confirmed that the prediction accuracy of fiber length for different environments or years and suggested that the prediction accuracy of fiber length using the *GFL* genes was largely consistent across environments or years at the late generations of progeny for plant breeding.

**TABLE 1:** Prediction accuracies of fiber length for different generations or years using the two datasets of the selected *GFL* genes for GBB collected in 2011, individually: **(A)** The RKHS model was used for the prediction and **(B)** The SVMRBF model was used for the prediction.

**Year**	**Generation**	**(A) 226 *GFL* SNPs/InDels as markers**		**(B) Expression of 125 selected *GFL* genes**	
		***r***	***P*-value**	***r***	***P*-value**
2011	F_9_	0.7830	0.00E + 00	0.7872	0.00E + 00
2010	F_8_	0.8334	0.00E + 00	0.7761	0.00E + 00
2009	F_7_	0.6719	0.00E + 00	0.6515	0.00E + 00

*The observed fiber lengths measured in 2010 or 2011 were the means of three replicates, while the observed fiber length measured in 2009 was from only one five-plant plot with no replicate, largely explaining the lower prediction accuracy of fiber length in 2009.*

### The Proper Training Population Size for Accurate Prediction of Fiber Length Using the *GFL* Genes

**FIGURE 6:**
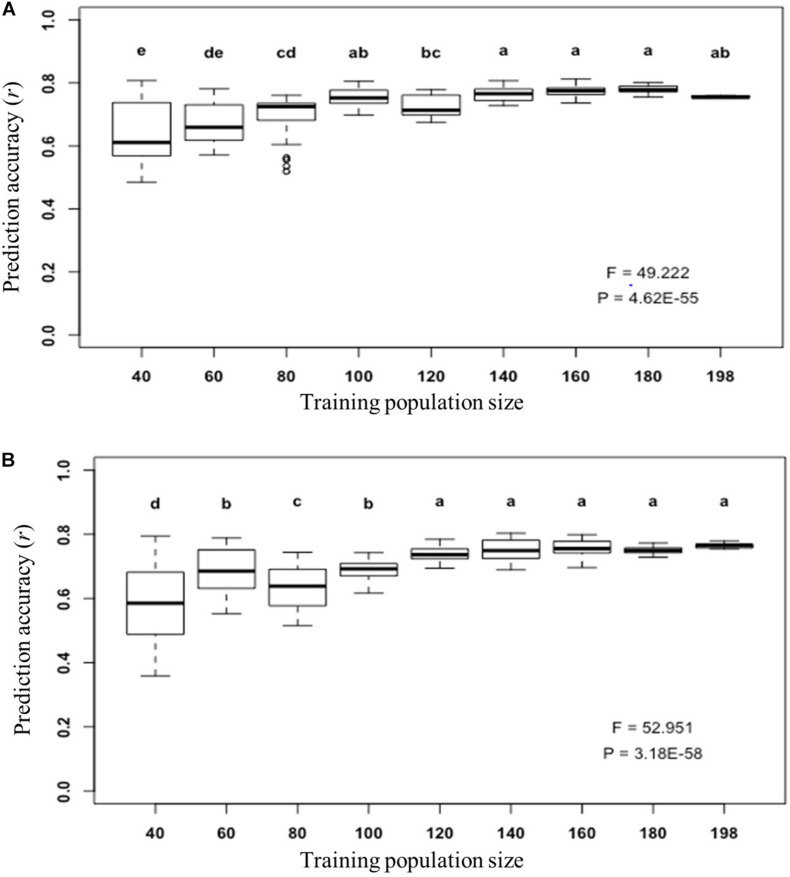
Prediction of fiber length with the selected *GFL* genes for GBB using different training population sizes. **(A)** Prediction of fiber length using the transcript expression abundances of the 125 selected *GFL* genes and the SVMRBF model (Figures 5A,B). **(B)** Prediction of fiber length using the 226 selected *GFL* SNPs/InDels as DNA markers and the RKHS model (Figure 4C). The prediction was carried out for 100 replications. Each number of lines was sampled for 10 times by bootstrap sampling, with each number sample being tested with 10 replications. Different letters, significant at CI ≥ 95%; same letter, not significant at CI ≥ 95%; error bar, standard deviation.

Furthermore, we determined what was the appropriate training population size to train a prediction model for fiber length prediction using the *GFL* genes by using their expression abundances (Figure 6A) and their SNPs/InDels as DNA markers (Figure 6B), individually. This is because the training population size is regarded to prediction accuracy and also to the cost for prediction model training. The populations consisting of a series of numbers of lines, from 40 to 198, were used to predict the fiber length using the selected optimal prediction models. Although the variation of the prediction accuracy increased as the training population size decreased, the prediction accuracy of the *GFL* genes for fiber length plateaued, when 100 lines were used, with the expressions of the 125 key *GFL* genes (Figure 6A). For prediction of fiber length using the 226 SNPs/InDels of the 226 SNP/InDel-containing *GFL* genes as DNA markers, the prediction accuracy of fiber length plateaued, when 120 lines were used (Figure 6B). Therefore, a training population size of 100–120 lines seemed proper to train a prediction model for accurate prediction of fiber length for progeny selection using either genotypes or expressions of the *GFL* genes.

## Discussion

One of the most important aims of molecular and genomic research is to develop molecular technologies that can enhance breeding in crop plants and livestock, and enhance medicine in humans. This study has demonstrated that the phenotype of a quantitative trait can be accurately predicted using the genes controlling the trait. The prediction accuracy of the cotton fiber length, which is used as the objective trait in this study, has approached its plateaued accuracy, with an accuracy of *r* = 0.83 (*P* = 0.000) using either the SNPs/InDels of 226 of the 474 *GFL* genes or the expressions of the 474 *GFL* genes. This prediction accuracy is as accurate as the prediction accuracy of maize grain yield (*r* = 0.85, *P* = 0.000), which is one of the most complex quantitative traits, using the maize grain yield (*ZmINGY*) genes (Zhang et al., 2020a). Moreover, the cotton fiber lengths predicted using these two genic datasets of the *GFL* genes are significantly correlated (*r* = 0.892, *P* = 0.000), further verifying the prediction accuracy of fiber length. The prediction accuracy of fiber length achieved using its contributing genes are 4%–315%, with an average of 95%, higher than those of *r* = 0.20–0.80 achieved for different quantitative traits using genome-wide DNA markers, genome-wide gene expressions, or genome-wide metabolites consisting of thousands to tens of thousands of omic features (Meuwissen et al., 2001; Lee et al., 2008; Crossa et al., 2010, 2013; De Los Campos et al., 2010b; Heffner et al., 2011a,b; Daetwyler et al., 2012; González-Camacho et al., 2012; Gouy et al., 2013; Morota et al., 2014; Speed and Balding, 2014; Xu et al., 2014, 2016; Beyene et al., 2015; Dan et al., 2016; Weissbrod et al., 2016; Islam et al., 2020). If the same species (cotton), same trait (fiber length, UHML), same prediction models (BayesB, GBLUP and RKHS), and same cross-validation scheme are considered for the comparison, the prediction accuracy of the cotton fiber length using the 740 SNPs/InDels of the 226 *GFL* genes as DNA markers were *r* = 0.80, 0.80, and 0.82 (*P* = 0.000) for GBLUP, BayesB, and RKHS, respectively, in this study (Figure 4A). These prediction accuracies are 116% higher than those of the fiber length predicted using 6,292 genome-wide SNPs (Islam et al., 2020). Furthermore, the prediction accuracy of cotton fiber length using the *GFL* genes is highly consistent across years (environments), even though the weathers between the years were quite different, with 2011 having unusual weather. This result is consistent with that of Zhang et al. (2020a) who showed that the genes controlling maize grain yields consistently predicted the maize grain yield across diverse climates and across different eco-agricultural systems. Finally, 100–120 plants are sufficient to properly train a model for accurate prediction of fiber length using the *GFL* genes, thus significantly reducing the cost for training and validating a model for phenotype prediction of a quantitative trait (Islam et al., 2020). These results, therefore, indicate that the genes controlling a quantitative trait are capable of and desirable for accurate prediction of the phenotype of a quantitative trait for progeny selection.

Zhang et al. (2020a) first proposed gene-based breeding (GBB), based on the ability, utility, and efficiency of the maize grain yield genes for accurate prediction of maize grain yield. GBB is an innovative plant breeding method that makes full use of the genes controlling the objective trait(s) through the entire process of plant breeding, including parent selection, cross design, and progeny selection. Three genic datasets of the genes are used for GBB individually or jointly: (i) the number of their favorable alleles (NFAs), (ii) their SNPs/InDels as DNA markers, and (iii) their expression abundances and networks. The results of this study that used two of the genic datasets for GBB provide a strong support for development and application of GBB for enhanced and accelerated plant breeding. Because the datasets of genes controlling the objective trait(s) are used for the entire breeding process, GBB allows not only accurately selecting for the progeny that are the most high-yielding, high-quality and highly resistant to biotic and abiotic stresses, but also accurately selecting the most desirable breeding materials or parents to approach the breeding objectives and wisely designing crosses that maximally combine the favorable alleles and heterotic genotypes of the genes controlling the objective trait(s) from the breeding materials into progeny. Therefore, GBB sheds great light on substantial and continued crop improvement, thus promising to help feed the world.

The findings of this study are achieved using cotton fiber length as the objective trait; nevertheless, the concepts and methods developed in this study are applicable to accurate prediction of other quantitative traits in crop plants, livestock, and humans, to development of GBB for enhanced crop and livestock improvement, and to development of gene-based medicine for enhanced human disease prevention, diagnosis and medicine. This conclusion is supported not only by the results of this study, but also by Zhang et al. (2020a) who accurately predicted the phenotype of grain yield in maize within and across diverse environments (locations). However, concerns may exist for practical use of the trait contributing genes in phenotype prediction of quantitative traits. The first concern may be genome-wide high-throughput cloning of the genes controlling an objective quantitative trait. We previously invented an innovative technology and developed an associated pipeline for genome-wide high-throughput cloning of the genes controlling quantitative traits and used it to have successfully cloned the 1,501 *ZmINGY* genes used by Zhang et al. (2020a) and the 474 *GFL* genes used for this study. Both the accurate prediction of cotton fiber length using the *GFL* genes (this study) and the accurate prediction of maize grain yield using the *ZmINGY* genes (Zhang et al., 2020a) consistently indicated that our novel gene cloning technology enables to genome-wide, high-throughput, and reliably clone the genes controlling quantitative traits. Because its gene cloning throughput, efficiency, and reliability are independent of the genome size, complexity, ploidy level, and availability of genomic knowledge and resources of a species, our gene cloning technology is applicable to genome-wide high-throughput cloning of genes controlling a quantitative trait in any species, including plants, animals, humans, and microbes. This technology and associated pipeline will be published and made available to the public soon.

The second concern may be variation of gene expression across environments. First, gene expression is the determinant of phenotype of a trait that results from interaction of numerous factors, including gene effects (additive and dominant), gene mutation, gene x gene interaction (epistasis), gene x genetic background or non-gene element interaction, epigenetic factors, and G x E interaction; therefore, it is a desirable type of omics for omics-based prediction of phenotypes. This study and Zhang et al. (2020a, b) revealed that the variation of a quantitative trait, such as cotton fiber length, maize grain yield, and ginseng ginsenoside content (Zhang et al., 2020b), is contributed by not only gene mutation, such as SNPs/InDels, but also by variation of gene expression. Therefore, the expression abundances of genes controlling the objective quantitative trait accurately predicted the phenotype of the fiber length in this study and the phenotype of the maize grain yield by Zhang et al. (2020a). Moreover, Zhang et al. (2019) conducted an extensive study on the variation of gene expression across environments and showed that that gene transcript expressions were highly consistent and highly reproducible across plants growing within a field trial replicate, between field trial replicates, and sampled from different years/locations (*r* = 0.90–0.98, *P* = 0.000). In addition, we recently showed that the phenotypic performance of offspring could be also accurately predicted using the expression abundances of genes related to the objective trait (grain yield) in parents in maize across very diverse climates, across eco-agricultural systems, and across populations (MZ, Y-HL, Y Wang, CF Scheuring, X Qi, J Pekar, SC Murray, W Xu, S-HS, H-BZ, submitted). These results together consistently indicate that the expression abundances of the genes contributing to the objective trait could predict the phenotype of the trait across environments, including different years, different climates, and different eco-agricultural systems, and across populations.

## Data Availability Statement

All datasets generated for this study are included in the article or Supplementary Material. The sequences of the 474 *GFL* genes can be found in NCBI GenBank under accession numbers: MW082098-MW082571.

## Author Contributions

H-BZ conceived, designed, and supervised the entire project. SX supervised the prediction of fiber length with the *GFL* genes using the nine prediction models. Y-HL performed the experiments and data analysis. YX and YC performed the fiber length prediction with the *GFL* genes using the prediction models. MZ helped with the data analysis and prepared the manuscript. CWS developed the RIL population, helped conduct the field trials, and phenotyped the fiber length. S-HS genotyped the SNPs or InDels of the *GFL* genes in the cotton population and parents. All authors contributed to the article and approved the submitted version.

## Conflict of Interest

The authors declare that the research was conducted in the absence of any commercial or financial relationships that could be construed as a potential conflict of interest.

## Supplementary Material

Supplementary Figure 1 Examples of validation of cotton *GFL* SNPs by allele-specific PCR.

Supplementary Figure 2 Selection of key *GFL* genes for GBB.

Supplementary Figure 3 Prediction of fiber length using different numbers of the effect-selected *GFL* genes with nine prediction models.

Supplementary Figure 4 Correlation of predicted fiber lengths between the 125 selected *GFL* genes and all 474 *GFL* genes.

Supplementary Table 1 Selection of the key *GFL* genes for GBB, according to their effects on fiber length (A),their roles in the *GFL* network (B), or the effects of their SNP/InDel mutations on fiber length (C).

Supplementary Table 2 Published cotton fiber length genes cloned by the traditional gene cloning methods and used as the positive control in this study.

Supplementary Table 3 The transcript sequences of the published cotton fiber length genes used as the positive control in this study.

Supplementary Table 4 Expression profile variation of the 474 *GFL* genes, presented in TPM (transcripts per million), in 10-dpa developing fibers of the cotton RIL population.

Supplementary Table 5 Prediction accuracy of fiber length with different numbers of randomly-selected *GFL* genes and randomly-selected unknown non-474 *GFL* cotton genes using nine prediction models.

Supplementary Table 6 Genotypes of all 740 SNPs/InDels contained in 226 *GFL* genes for prediction of fiber length.

Supplementary Table 7 Genotypes of 226 SNPs/InDels contained in 226 *GFL* genes, with only one SNP or InDel per gene, for prediction of fiber length.

Supplementary Table 8 The 125 key *GFL* genes selected for GBB.
