# Supplementary material for: Accurate Prediction of a Quantitative Trait Using the Genes Controlling the Trait for Gene-Based Breeding in Cotton
Source: Front Plant Sci. 2020 Nov 9;11:583277. doi: 10.3389/fpls.2020.583277 (PMC7690289; doi:10.3389/fpls.2020.583277)
Supplement: Supplementary Figure 1 — Examples of validation of cotton GFL SNPs by allele-specific PCR. [file Data_Sheet_1.PDF]

Supplementary Material for the Article:

Accurate prediction of a quantitative trait using the genes controlling the trait for gene-based breeding in cotton

Yun-Hua Liu[1], Yang Xu[2], Meiping Zhang[1], Yanru Cui[2], Sing-Hoi Sze[3], C. Wayne Smith[1], Shizhong Xu[2,*] and Hong-Bin Zhang[1,*]

[1] Department of Soil and Crop Sciences, Texas A&M University, College Station, TX 77843, USA

[2] Botany and Plant Sciences, University of California, Riverside, CA 92521, USA

[3] Department of Computer Science and Engineering and Department of Biochemistry and Biophysics, Texas A&M University, College Station, TX 77843, USA

Y.-H.L., Y.X. and M.Z. contributed equally to this study.

[*]Correspondence (H.-B.Z: Tel +1-979-862-2244; fax +1-979-845-0456; email hbz7049@tamu.edu. S.X: Tel 1-951-827-5898; fax +1-951-827-4437; email: shizhong.xu@ucr.edu)

Supplementary Material: Supplementary References = 18; Supplementary Figures (.pptx) = 4; Supplementary Tables (.xlsx) = 8.

Supplementary References cited for Supplementary Table S2:

Abdurakhmonov IY, Buriev ZT, Saha S, Jenkins JN, Abdukarimov A, Pepper AE (2014) Phytochrome RNAi enhances major fibre quality and agronomic traits of the cotton *Gossypium hirsutum* L. Nat Commun 5:3062.

Han LB, Li YB, Wang HY, Wu XM, Li CL, Luo M, Wu SJ, et al. (2013) The dual functions of *WLIM1a* in cell elongation and secondary wall formation in developing cotton fibers. Plant Cell 25:4421–4438.

Hao J, Tu L, Hu H, Tan J, Deng F, Tang W, Nie Y, Zhang X (2012) *GbTCP*, a cotton TCP transcription factor, confers fibre elongation and root hair development by a complex regulating system. J Exp Bot 63:6267–6281.

Huang GQ, Gong SY, Xu WL, Li W, Li P, Zhang CJ, Li DD, et al. (2013) A fasciclin-like arabinogalactan protein, *GhFLA1*, is involved in fiber initiation and elongation of cotton. Plant Physiol 161:1278–1290.

Jiang Y, Guo W, Zhu H, Ruan YL, Zhang T (2012) Overexpression of *GhSusA1* increases plant biomass and improves cotton fiber yield and quality. Plant Biotech J 10:301–312.

Lee J, Burns TH, Light G, Sun Y, Fokar M, Kasukabe Y, Fujisawa.K, Maekawa Y, Allen RD (2010) *Xyloglucan endotransglycosylase/hydrolase* genes in cotton and their role in fiber elongation. Planta 232:1191–1205.

Li X-B, Fan X-P, Wang X-L, Cai L, Yang W-C (2005) The cotton *ACTIN1* gene is functionally expressed in fibers and participates in fiber elongation. Plant Cell 17:859–875.

Li Y, Liu D, Tu L, Zhang X, Wang L, Zhu L, Tan J, Deng F (2010) Suppression of *GhAGP4* gene expression repressed the initiation and elongation of cotton fiber. Plant Cell Rep 29:193–202.

Luo M, Xiao Y, Li X, Lu X, Deng W, Li D, Hou L, et al. (2007) *GhDET2*, a steroid 5a-reductase, plays an important role in cotton fiber cell initiation and elongation. Plant J 51:419–430.

Machado A, Wu Y, Yang Y, Llewellyn DJ, Dennis ES (2009) The MYB transcription factor *GhMYB25* regulates early fibre and trichrome development. Plant J 59:52–62.

Pu L, Li Q, Fan X, Yang W, Xue Y (2008) The R2R3 MYB transcription factor *GhMYB109* is required for cotton fiber development. Genetics 180:811–820.

Tan J, Tu L, Deng F, Hu H, Nie Y, Zhang X (2013) A genetic and metabolic analysis revealed that cotton fiber cell development was retarded by flavonoid naringenin. Plant Physiol 162:86–95.

Wang HY, Wang J, Gao P, Jiao GL, Zhao PM, Li Y, Wang GL, Xia GX (2009) Down-regulation of *GhADF1* gene expression affects cotton fibre properties. Plant Biotech J 7:13–23.

Wang H, Guo Y, Lv F, Zhu H, Wu S, Jiang Y, Li F, et al. (2010a) The essential role of *GhPEL* gene, encoding a pectate lyase, in cell wall loosening by depolymerization of the de-esterified pectin during fiber elongation in cotton. Plant Mol Biol 72:397–406.

Wang J, Wang HY, Zhao PM, Han LB, Jiao GL, Zheng YY, Huang SJ, Xia GX (2010b) Overexpression of a profilin (*GhPFN2*) promotes the progression of developmental phases in cotton fibers. Plant Cell Physiol 51:1276–1290.

Wang L, Li XR, Lian H, Ni DA, He YK, Chen XY, Ruan YL (2010c) Evidence that high activity of vacuolar invertase is required for cotton fiber and *Arabidopsis* root elongation through osmotic dependent and independent pathways, respectively. Plant Physiol 154:744–756.

Xiao YH, Li DM, Yin MH, Li XB, Zhang M, Wang YJ, Dong J, et al. (2010) Gibberellin 20-oxidase promotes initiation and elongation of cotton fibers by regulating gibberellin synthesis. J Plant Physiol 167:829–837.

Xu B, Gou JY, Li FG, Shangguan XX, Zhao B, Yang CQ, Wang LJ, et al. (2013) A cotton BURP domain protein interacts with α-expansin and their co-expression promotes plant growth and fruit production. Mol Plant 6:945–958.

Supplementary Material

FIGURE S1. Examples of validation of cotton *GFL* SNPs by allele-specific PCR**.** The suffix of each *GFL* gene name indicates the position of the SNP in its sequence. The genomic DNA of cotton TAM 94L-25 (1), NMSI 1331 (2), 15QQ-15 (3) and 15QQ-29 (4) were used as templates, amplified by PCR using a forward allelic primer with or without a sequence tag and reverse primer, and fractionated on 2% agarose gels. F-tag, forward allelic primers with a 21-nucleotide tag; F, forward allelic primer with no tag; R, reverse primer. Sequence analysis showed that the SNPs in *GFL472-357*, *GFL048-413* and *GFL044-131* were in heterozygous states in TAM 94L-25 and NMSI 1331, whereas 15QQ-15 and 15QQ-29 were F_1 hybrids derived from *G. hirsutum* x *G barbadense* crosses, 13P-54//11K-13/Del Cerro and 13P-54//11K-13/NMSI 1331, respectively.

FIGURE S2. Selection of key *GFL* genes for GBB. The selection was performed based on their roles in the *GFL* network, their contributions to fiber length and their SNP/InDel mutations. Gene expression profiles were used for the prediction. Different letters, significant at CI ≥ 95%; same letter, not significant at CI ≥ 95%; error bar, standard deviation for 10 replications. **(A)** Prediction accuracy of fiber length with the 19 network-edge key *GFL* genes (**Supplementary Table S1B**). I, The 19 *GFL* genes that had edge number variation in the *GFL* network significantly influencing fiber length; II, 19 randomly-selected *GFL* genes**. (B)** Prediction accuracy of fiber length with the 226 SNP/InDel-containing *GFL* genes (**Supplementary Table S1C**). I, The 226 SNP/InDel-containing *GFL* genes; II, 226 randomly-selected *GFL* genes. **(C)** Prediction accuracy of fiber length using a subset of 226 *GFL* genes selected according to their effects on fiber length (**Supplementary Table S1A**). X, a subset of 226 *GFL* genes consisting of all 54 *GFL* genes having the positive effects, 59 *GFL* genes having the smallest negative effects and 113 *GFL* genes having the largest negative effects on fiber length; Y, a subset of 226 *GFL* genes consisting of all 54 *GFL* genes having the positive effects and 172 *GFL* genes having the smallest negative effects on fiber length; W, 226 randomly-selected *GFL* genes; Z, a subset of 226 *GFL* genes having the largest negative effects on fiber length.

FIGURE S3. Prediction of fiber length using different numbers of the effect-selected *GFL* genes with nine prediction models. The transcript expressions of the *GFL* genes were used for the prediction. Each number of the selected *GFL* genes consisted of 50% that had the positive effects and smallest negative effects on fiber length and 50% that had the largest negative effect on fiber length. Different colors indicate the different numbers of the effect-selected *GFL* genes used for the prediction of fiber length.

FIGURE S4. Correlation of predicted fiber lengths between the 125 selected *GFL* genes and all 474 *GFL* genes. The expression profiles of these genes and the optimal model for each set of the *GFL* genes were used for the prediction.

TABLE S1. Selection of the key *GFL* genes for GBB, according to their effects on fiber length (A), the effects of their SNP/InDel mutations on fiber length (B), and/or their roles in the *GFL* network (C).

TABLE S2. Published cotton fiber length genes cloned by the traditional gene cloning methods and used as the positive control in this study.

TABLE S3. The transcript sequences of the published cotton fiber length genes used as the positive control in this study.

TABLE S4. Expression profile variation of the 474 *GFL* genes, presented in TPM (transcripts per million), in 10-dpa developing fibers of the cotton RIL population.

TABLE S5. Prediction accuracy of fiber length with different numbers of randomly-selected *GFL* genes and randomly-selected unknown non-474 *GFL* cotton genes using nine prediction models.

TABLE S6. Genotypes of all 740 SNPs/InDels contained in 226 *GFL* genes for prediction of fiber length.

TABLE S7. Genotypes of 226 SNPs/InDels contained in 226 GFL genes, with only one SNP or InDel per gene, for prediction of fiber length.

TABLE S8. The 125 key *GFL* genes selected for GBB.
