# Supplementary material for: Accurate Prediction of a Quantitative Trait Using the Genes Controlling the Trait for Gene-Based Breeding in Cotton
Source: Front Plant Sci. 2020 Nov 9;11:583277. doi: 10.3389/fpls.2020.583277 (PMC7690289; doi:10.3389/fpls.2020.583277)
Supplement: Supplementary file 10 [file Presentation_1.PPTX]

## Slide 1
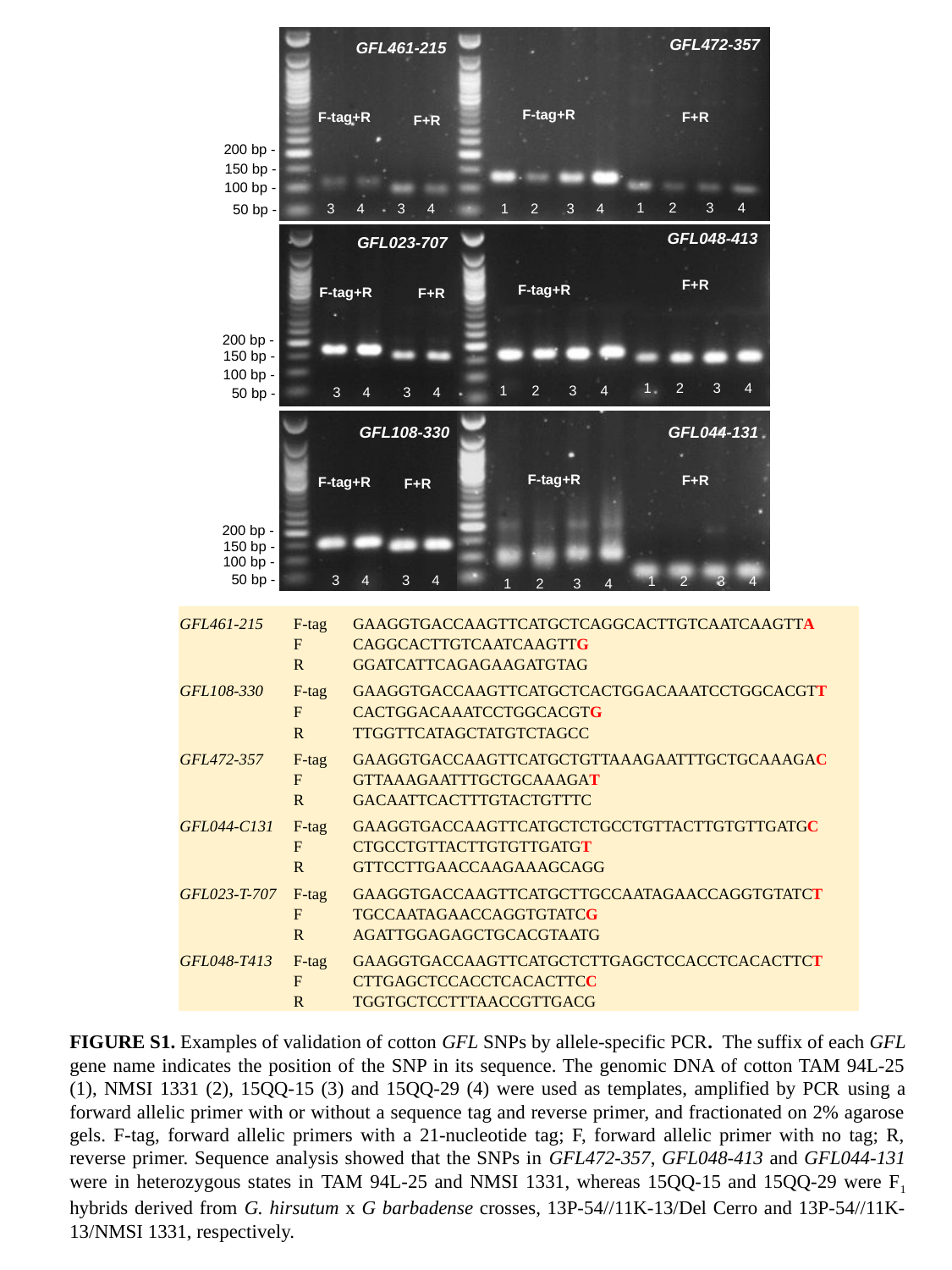

GFL472-357
GFL461-215
GFL048-413
GFL023-707
GFL044-131
GFL108-330
F-tag+R
F-tag+R
F+R
F+R
200 bp -
150 bp -
100 bp -
1
2
3
4
1
2
3
4
3
4
3
4
50 bp -
F+R
F-tag+R
F-tag+R
F+R
200 bp -
150 bp -
100 bp -
1
2
3
4
1
2
3
4
3
4
3
4
50 bp -
F-tag+R
F+R
F-tag+R
F+R
200 bp -
150 bp -
100 bp -
50 bp -
3
4
3
4
1
2
3
4
1
2
3
4
| GFL461-215 | F-tag | GAAGGTGACCAAGTTCATGCTCAGGCACTTGTCAATCAAGTTA |
| --- | --- | --- |
| | F | CAGGCACTTGTCAATCAAGTTG |
| | R | GGATCATTCAGAGAAGATGTAG |
| GFL108-330 | F-tag | GAAGGTGACCAAGTTCATGCTCACTGGACAAATCCTGGCACGTT |
| | F | CACTGGACAAATCCTGGCACGTG |
| | R | TTGGTTCATAGCTATGTCTAGCC |
| GFL472-357 | F-tag | GAAGGTGACCAAGTTCATGCTGTTAAAGAATTTGCTGCAAAGAC |
| | F | GTTAAAGAATTTGCTGCAAAGAT |
| | R | GACAATTCACTTTGTACTGTTTC |
| GFL044-C131 | F-tag | GAAGGTGACCAAGTTCATGCTCTGCCTGTTACTTGTGTTGATGC |
| | F | CTGCCTGTTACTTGTGTTGATGT |
| | R | GTTCCTTGAACCAAGAAAGCAGG |
| GFL023-T-707 | F-tag | GAAGGTGACCAAGTTCATGCTTGCCAATAGAACCAGGTGTATCT |
| | F | TGCCAATAGAACCAGGTGTATCG |
| | R | AGATTGGAGAGCTGCACGTAATG |
| GFL048-T413 | F-tag | GAAGGTGACCAAGTTCATGCTCTTGAGCTCCACCTCACACTTCT |
| | F | CTTGAGCTCCACCTCACACTTCC |
| | R | TGGTGCTCCTTTAACCGTTGACG |
FIGURE S1. Examples of validation of cotton GFL SNPs by allele-specific PCR. The suffix of each GFL gene name indicates the position of the SNP in its sequence. The genomic DNA of cotton TAM 94L-25 (1), NMSI 1331 (2), 15QQ-15 (3) and 15QQ-29 (4) were used as templates, amplified by PCR using a forward allelic primer with or without a sequence tag and reverse primer, and fractionated on 2% agarose gels. F-tag, forward allelic primers with a 21-nucleotide tag; F, forward allelic primer with no tag; R, reverse primer. Sequence analysis showed that the SNPs in GFL472-357, GFL048-413 and GFL044-131 were in heterozygous states in TAM 94L-25 and NMSI 1331, whereas 15QQ-15 and 15QQ-29 were F1 hybrids derived from G. hirsutum x G barbadense crosses, 13P-54//11K-13/Del Cerro and 13P-54//11K-13/NMSI 1331, respectively.
