# Supplementary material for: Accurate Prediction of a Quantitative Trait Using the Genes Controlling the Trait for Gene-Based Breeding in Cotton
Source: Front Plant Sci. 2020 Nov 9;11:583277. doi: 10.3389/fpls.2020.583277 (PMC7690289; doi:10.3389/fpls.2020.583277)
Supplement: Supplementary file 11 [file Presentation_2.PPTX]

## Slide 1
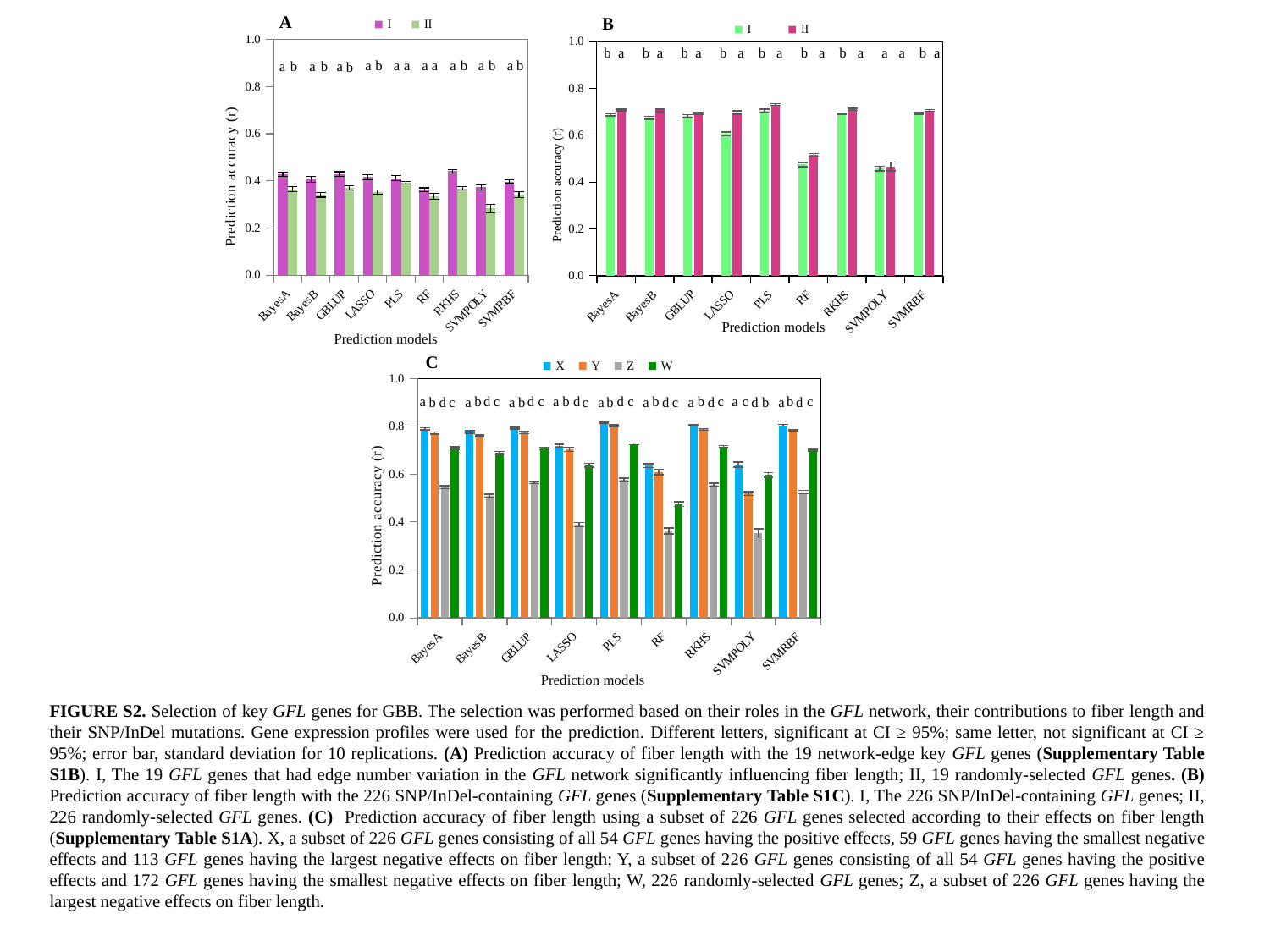

A
### Chart
| Category | | |
|---|---|---|
| BayesA | 0.426874107669727 | 0.363787720107393 |
| BayesB | 0.405520777 | 0.340868813 |
| GBLUP | 0.428727526331636 | 0.370839182551901 |
| LASSO | 0.415042271 | 0.351492476 |
| PLS | 0.410868409378894 | 0.391256135668096 |
| RF | 0.360974155 | 0.33449301 |
| RKHS | 0.440697713349222 | 0.367587782899895 |
| SVMPOLY | 0.372128823214521 | 0.282419353111114 |
| SVMRBF | 0.396096776272979 | 0.340705231487478 |a
a
a
a
a
a
b
b
a
b
a
b
a
b
a
b
a
b
Prediction models
### Chart
| Category | | |
|---|---|---|
| BayesA | 0.687172144690383 | 0.707893868201314 |
| BayesB | 0.673267329412902 | 0.706364102089954 |
| GBLUP | 0.681198737425736 | 0.693904445049343 |
| LASSO | 0.605623438878305 | 0.697122288938349 |
| PLS | 0.705042630280979 | 0.730893369061822 |
| RF | 0.474837265151097 | 0.516515759032756 |
| RKHS | 0.692617465721179 | 0.710824028310229 |
| SVMPOLY | 0.457616812183677 | 0.465958702000399 |
| SVMRBF | 0.692192429846103 | 0.705407023194258 |B
 b a b a b a b a b a b a b a a a b a
Prediction models
### Chart
| Category | | | | |
|---|---|---|---|---|
| BayesA | 0.79044931771829 | 0.772405736864356 | 0.545799009269834 | 0.710011134466532 |
| BayesB | 0.777478096426762 | 0.760588249556144 | 0.50990815174352 | 0.689641219496524 |
| GBLUP | 0.792884634676262 | 0.77465216651613 | 0.566354346300835 | 0.707972403262785 |
| LASSO | 0.717908174700324 | 0.703039928147164 | 0.389058031947674 | 0.637800632832013 |
| PLS | 0.816170630531161 | 0.802932450092213 | 0.5778139256103 | 0.727201190929418 |
| RF | 0.635800292610375 | 0.609123262946581 | 0.36224846701487 | 0.475241660053247 |
| RKHS | 0.80432748325483 | 0.787924841493139 | 0.555531944119126 | 0.715909093704399 |
| SVMPOLY | 0.64070370495743 | 0.519738545577257 | 0.3552039516834 | 0.597125861388604 |
| SVMRBF | 0.80444358232583 | 0.782400543695496 | 0.525385547736796 | 0.700893125985843 |C
c
d
c
a
b
b
c
a
d
c
d
b
a
c
c
d
b
b
b
a
c
d
c
a
d
a
a
a
c
b
d
b
a
b
d
d
Prediction models
FIGURE S2. Selection of key GFL genes for GBB. The selection was performed based on their roles in the GFL network, their contributions to fiber length and their SNP/InDel mutations. Gene expression profiles were used for the prediction. Different letters, significant at CI ≥ 95%; same letter, not significant at CI ≥ 95%; error bar, standard deviation for 10 replications. (A) Prediction accuracy of fiber length with the 19 network-edge key GFL genes (Supplementary Table S1B). I, The 19 GFL genes that had edge number variation in the GFL network significantly influencing fiber length; II, 19 randomly-selected GFL genes. (B) Prediction accuracy of fiber length with the 226 SNP/InDel-containing GFL genes (Supplementary Table S1C). I, The 226 SNP/InDel-containing GFL genes; II, 226 randomly-selected GFL genes. (C) Prediction accuracy of fiber length using a subset of 226 GFL genes selected according to their effects on fiber length (Supplementary Table S1A). X, a subset of 226 GFL genes consisting of all 54 GFL genes having the positive effects, 59 GFL genes having the smallest negative effects and 113 GFL genes having the largest negative effects on fiber length; Y, a subset of 226 GFL genes consisting of all 54 GFL genes having the positive effects and 172 GFL genes having the smallest negative effects on fiber length; W, 226 randomly-selected GFL genes; Z, a subset of 226 GFL genes having the largest negative effects on fiber length.
