# Supplementary material for: Accurate Prediction of a Quantitative Trait Using the Genes Controlling the Trait for Gene-Based Breeding in Cotton
Source: Front Plant Sci. 2020 Nov 9;11:583277. doi: 10.3389/fpls.2020.583277 (PMC7690289; doi:10.3389/fpls.2020.583277)
Supplement: Supplementary file 12 [file Presentation_3.PPTX]

## Slide 1
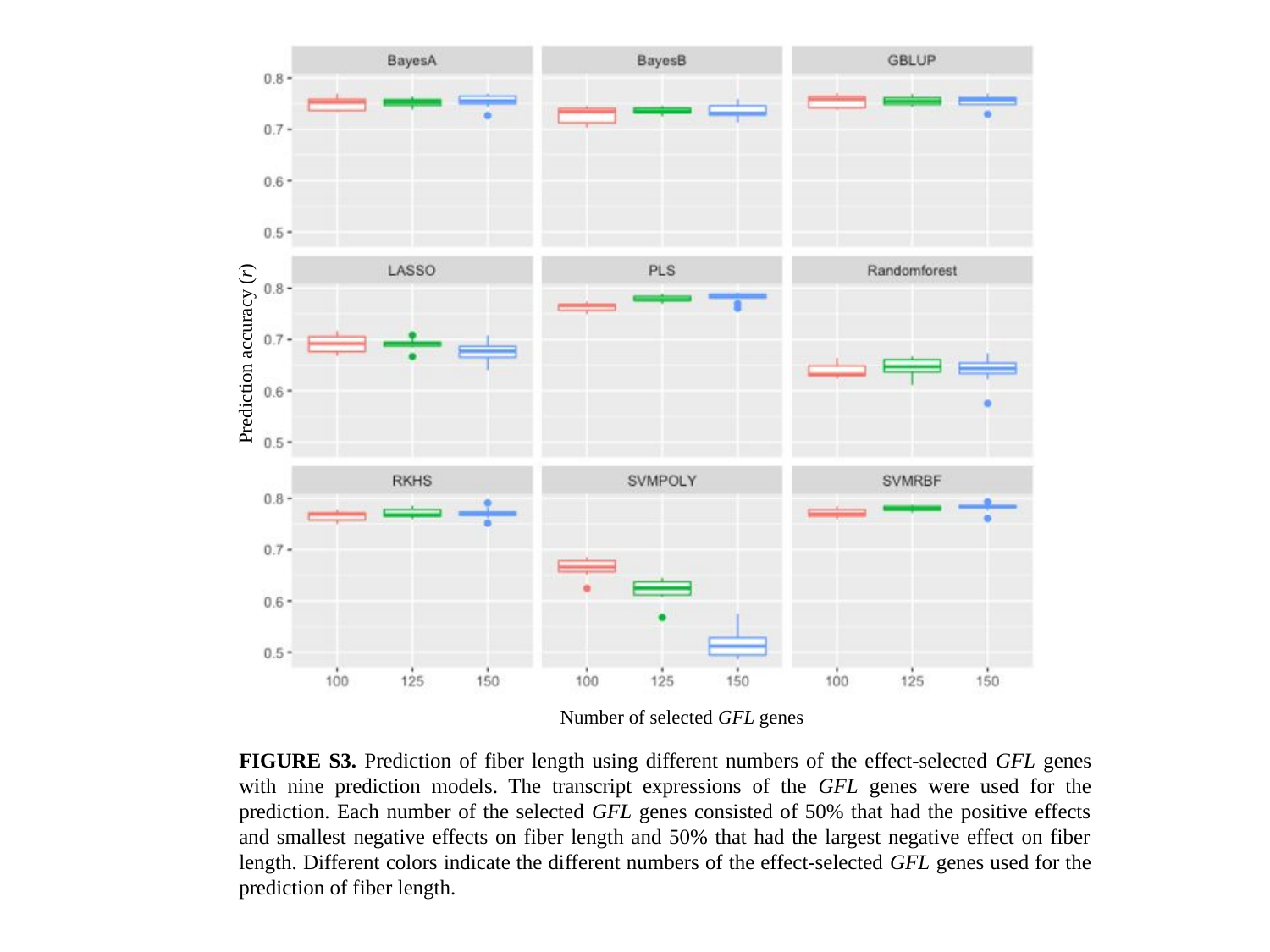

Prediction accuracy (r)
Number of selected GFL genes
FIGURE S3. Prediction of fiber length using different numbers of the effect-selected GFL genes with nine prediction models. The transcript expressions of the GFL genes were used for the prediction. Each number of the selected GFL genes consisted of 50% that had the positive effects and smallest negative effects on fiber length and 50% that had the largest negative effect on fiber length. Different colors indicate the different numbers of the effect-selected GFL genes used for the prediction of fiber length.
