# Supplementary material for: Accurate Prediction of a Quantitative Trait Using the Genes Controlling the Trait for Gene-Based Breeding in Cotton
Source: Front Plant Sci. 2020 Nov 9;11:583277. doi: 10.3389/fpls.2020.583277 (PMC7690289; doi:10.3389/fpls.2020.583277)
Supplement: Supplementary file 13 [file Presentation_4.PPTX]

## Slide 1
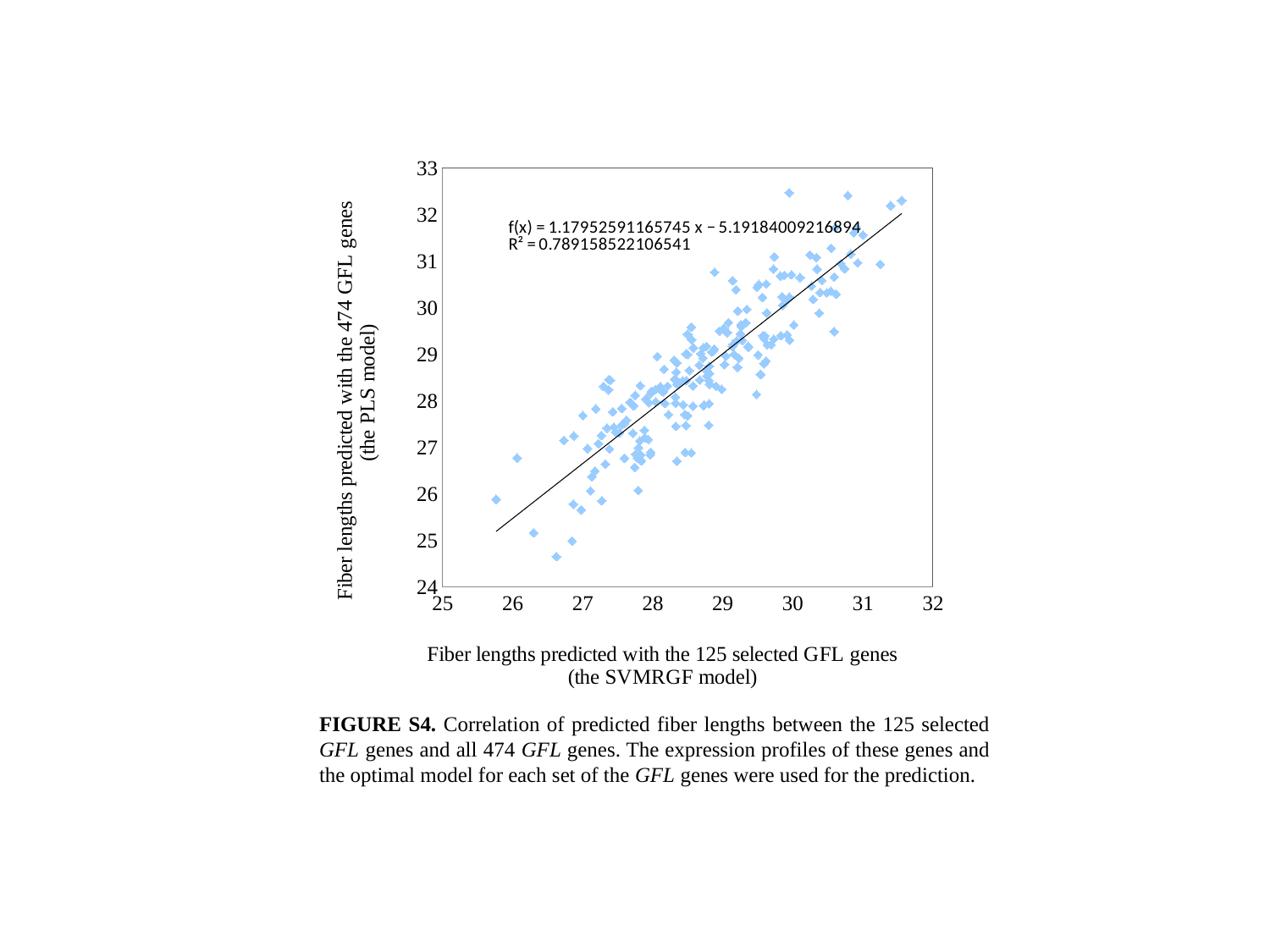

### Chart
| Category | yp2_474GFL_GEPS_PLS |
|---|---|FIGURE S4. Correlation of predicted fiber lengths between the 125 selected GFL genes and all 474 GFL genes. The expression profiles of these genes and the optimal model for each set of the GFL genes were used for the prediction.
